# Serum Markers in Rheumatoid Arthritis: A Longitudinal Study of Patients Undergoing Infliximab Treatment

**DOI:** 10.1155/2015/276815

**Published:** 2015-12-16

**Authors:** Oddgeir Selaas, Hilde H. Nordal, Anne-Kristine Halse, Johan G. Brun, Roland Jonsson, Karl A. Brokstad

**Affiliations:** ^1^Broegelmann Research Laboratory, Department of Clinical Science, University of Bergen, 5020 Bergen, Norway; ^2^Department of Rheumatology, Haukeland University Hospital, University of Bergen, 5020 Bergen, Norway

## Abstract

*Objective.* The aim of this study was to investigate the clinical effect and serum markers in a cohort of rheumatoid arthritis patients with moderate to high disease activity, participating in an open clinical phase IV study conducted in Norway between 2001 and 2003 receiving infliximab treatment.* Method.* A total of 39 patients were studied, with a mean age of 54 years and 12-year disease duration. The analyses were performed using serum from patients at four assessment time points: baseline and 3, 6, and 12 months after starting treatment with infliximab. A wide variety of clinical data was collected and disease activity of 28 joints and Simple Disease Activity Index were calculated. The joint erosion was determined by X-ray imaging and the Sharp/van der Heijde score was determined. Serum analysis included multiplex immunoassays for 12 cytokines, 5 matrix metalloproteases, and 2 VEGFs.* Results.* The majority of the RA patients in this study had initially moderate to high disease activity and the infliximab treatment reduced the disease activity significantly and also reduced any further joint destruction and improved disease status. Most of the serum levels of cytokines and metalloproteases remained unchanged during the course of the study, and we were unable to detect changes in TNF-*α* in serum. Serum levels of IL-6 and VEGF-A decreased significantly after initiation of infliximab treatment.* Conclusion.* The serum levels of IL-6 and VEGF-A may be promising disease markers as they vary with disease progression. The clinical significance of these findings is yet to be determined and has to be confirmed in future clinical trials before being applied in the clinics.

## 1. Introduction

Rheumatoid arthritis (RA) is an autoimmune chronic inflammatory disease characterised by persistent synovitis and progressive erosion of the cartilage and the bone in joints, in addition to the presence of autoantibodies like rheumatoid factor (RF) and anti-citrullinated protein/peptide antibodies (ACPA). Left untreated RA can lead to severe damage of joints and immobility and significantly reduce the quality of life. The prevalence of RA is approximately 0.5–1% of the population and it affects women 2.5 times more frequently than men [1]. Genetic predisposition and smoking are known risk factors for RA, and diagnosis is based on several criteria from both clinical assessments and laboratory analyses. The combination of CRP and ESR and autoantibodies like RF and ACPA are frequently used in the assessment and diagnosis of RA patients [2–4]. In addition, a variety of clinical data are recorded to support the RA diagnosis, including number of tender and swollen joints, high-resolution X-ray of involved joints, evaluation of disease activity using a visual analogue scale, and utilizing mathematical algorithms to determine the overall disease activity like disease activity score of 28 joints (DAS28 [5]) or Simplified Disease Activity Index (SDAI [6, 7]). Clinical diagnosis of RA is based on classification criteria and guidelines from the American College of Rheumatology (ACR) and the European League Against Rheumatism (EULAR). Collaboration between the two resulted in 2010 in a revised set of the older 1987 criteria [8, 9]. These new classification criteria focus on patients with no prior RA diagnosis and seek to determine definite RA in the absence of alternative diagnoses.

Treating RA patients has traditionally been carried out using immunosuppressive drugs like corticosteroids, disease-modifying antirheumatic drugs (DMARDs) like methotrexate, and nonsteroidal anti-inflammatory drugs (NSAIDs). In the last 10–20 years biological DMARDs have been introduced in the treatment of RA. These consist of a variety of cytokine inhibitors and other immune modulators. Due to the high cost of biological DMARDs, it is very important to identify nonresponders at earliest time point, the development of anti-drug antibodies, and disease progression markers. The aim of this study was to evaluate the effect of infliximab treatment on a cohort of advanced RA patients over a one-year period and to identify serum markers that could have relevance to disease progression, remission, or prognostic value.

## 2. Material and Methods

### 2.1. Patients and Samples

A total of 39 patients (Tables 1 and 2) defined by classification criteria [9] were selected from a cohort of 76 patients participating in an open clinical phase IV study receiving the TNF-*α* inhibitor Remicade (infliximab). Patients were included between October 2001 and March 2003 and then followed from baseline and up to 3 years after inclusion [10]. The patients were given 3 mg/kg infliximab at weeks 0, 2, and 6 and then every 8th week concomitantly with methotrexate (11 mg/week) and daily prednisolone. The treatment and sample collection were carried out at three hospitals in Western Norway: Førde Central Hospital, Haukeland University Hospital, and Haugesund Rheumatism Hospital. Rheumatologist assessed the patients and a range of clinical data was recorded. The assessment time points were at baseline and 3, 6, and 12 months afterwards. From the cohort we selected only patients that provided complete sample sets from the chosen assessments points. Samples were stored immediately after collection at −80°C.

### 2.2. Approvals

This study was approved by the Regional Ethical Committee (REK II 84.01) and the Norwegian Medicines Agency. Informed and signed consent was obtained from all patients.

### 2.3. Disease Activity Scores

We have used two disease activity scores, namely, Disease Activity Score of 28 joints (DAS28) and Simplified Disease Activity Index (SDAI) [5–7]. These are validated scores that describe the overall disease activity and are calculated from both subjective and objective readings, number of swollen joints from 28 joints (SWJ28), number of tender joints from 28 joints (TEN28), C-reactive protein (CRP), erythrocyte sedimentation rate (ESR), patient's global assessment (PGA, pVAS), and physician's global assessment (MDGA, mdVAS). The DAS28 score (0 to 10) is described as follows: a score below 2.6 indicates that the patient is in remission. A DAS28 score from >2.6 to ≤3.2 and from >3.2 to ≤5.1 indicates low and moderate disease activity, respectively, and a DAS28 score above is indicative of high disease activity. SDAI, a simplified disease index, is also useful (0–100) when not all clinical data are available, with scores above 26 indicating high disease activity. Scores between 11 to 26 are moderate, from 3.3 to 11 show low disease activity, and below 3.3 are considered in remission.

### 2.4. Analyses of Cytokines and MMPs and VEGFs Levels in Serum

Kits based on the Luminex bead immunoassay technology were used to detect cytokines, matrix metalloproteases (MMPs), and vascular endothelial growth factors (VEGFs) in serum. In order to assay the 12 cytokines, IL-1*β*, IL-2, IL-4, IL-5, IL-6, IL-10, IL-12, IL-17A, IL-18, IFN-*γ*, TNF-*α*, and TNF-*β*, we mixed a ProcartaPlex premade multiplex-kit with single-plex kits (Cat. nos. EPX060-10009-901, EPX010-10243, EPX010-10221, EPX010-10215, EPX010-12017, EPX010-10267, and EPX010-10202 (Affymetrix, eBioscience, Inc., San Diego, USA)). MMP-1, MMP-7, MMP-8, MMP-12, and MMP-13 were assayed by using ProcartaPlex Human MMP-Panel I 5 plex (EPX050-10015-901, Affymetrix, eBioscience). VEGF-A and VEGF-D were measured using the single-plex assays EPX010-10277 and EPX010-12076 (Affymetrix), respectively. We followed the manufacturers recommended procedure for these assays using a Luminex 100 (Luminex Corp., Austin, TX, USA) and STarStation v.2 software (Applied Cytometry, Dinnington, UK).

### 2.5. Data Processing

All data were processed and analysed, and all graphs have been prepared in GraphPad Prism 6 (GraphPad Software Inc., La Jolla, USA).

## 3. Results

In this longitudinal study we have monitored clinical and serological data from a cohort of 39 RA patients receiving infliximab treatment over one year.

### 3.1. Disease Activity Scores (DAS)

**Figure 1:**
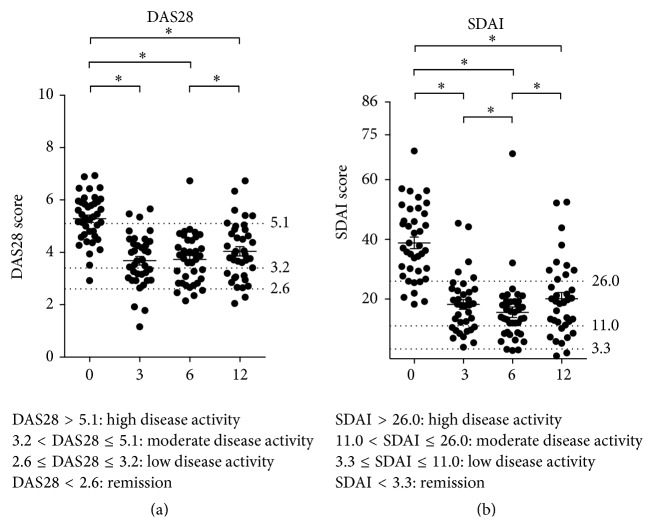
DAS28 and SDAI scores. Scatter dot plots where each dot represents one individual patient. (a) SDAI and (b) DAS28 score distribution during the study period. The *y*-axes represent the disease activity score. The horizontal lines in the dot plot indicated the mean value. The dotted horizontal lines indicate borders between categories of disease activity: (i) high; (ii) moderate; (iii) low; and (iv) patients who are considered in remission. The *x*-axis is the four assessment time points indicated as 0, 3, 6, and 12 months after treatment is initiated.

A majority of the patients were in the high disease activity group before treatment was started with an average DAS28 score of 5.3 ± 0.1 (Figure 1(a)). After onset of treatment, the majority of patients moved to low and moderate disease activity, with an average DAS28 score of 4.0 ± 0.2 after 12 months (*p* < 0.0001).

Similarly, the SDAI score showed that most patients were in the high disease activity group before treatment (Figure 1(b)) with an average SDAI score of 38.8 ± 2.0. During treatment the patients as a group moved to moderate and low disease activity with an average of SDAI score 20.0 ± 2.1 after 12 months (*p* < 0.0001).

### 3.2. X-Ray Assessment of Joints

**Figure 2:**
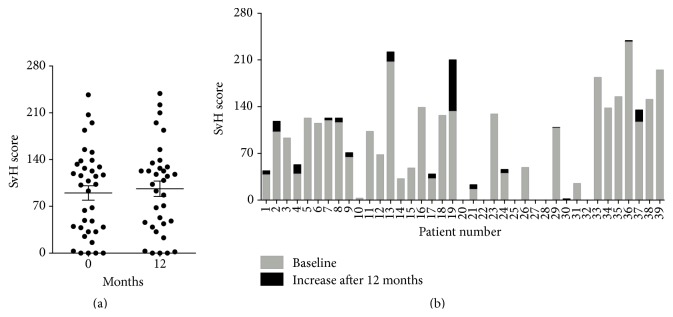
Progression of joint erosion. Erosions in the affected joints of the RA cohort, where the *y*-axes represent the Sharp/van der Heijde scores; (a) the *x*-axis illustrates the two assessment time points indicated as 0 and 12 months after treatment in a paired *t*-test; (b) the *x*-axis represents the patients' individual progression in joint erosion in a histogram, where the grey bars illustrate baseline joint erosions and the black bars illustrate additional joint erosions after 12 months.

The erosion of joints was scored according to Sharp/van der Heijde (SvH) [11]. The SvH scores were relatively stable during the study period (Figure 2(a)) with a mean score of 90.1 (± 10.8 SEM, range 0–237) and 96.4 (± 11.5 SEM, range 0–239), before and after 12 months with treatment, respectively. A few patients had a mild progression, and one patient had severe progression of joint erosion during the 12 months study period (Figure 2(b)).

### 3.3. Serological Markers

#### 3.3.1. Cytokines

**Figure 3:**
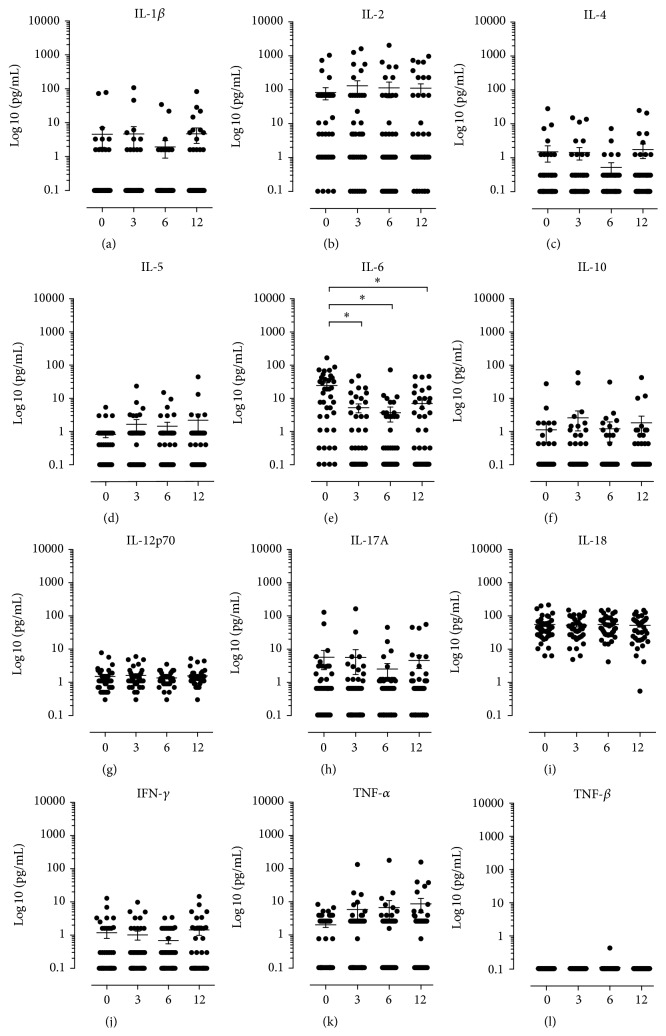
The serum cytokine levels in the rheumatoid arthritis patients. (a) to (l) are scatter dot plots of serum cytokine levels, where each dot represents one individual patient. The *x*-axes illustrate the four assessment months after infliximab treatment is initiated. The concentrations in serum of their respective cytokine in picograms per millilitre (pg/mL) on a log⁡10 scale. The horizontal lines in the dot plot illustrate the mean, and the vertical error lines represent ± standard error mean (SEM) in a paired *t*-test.

During the one-year study period, we monitored the levels of 12 common cytokines, representing a wide variety of immunological signalling functions (polarization). In general all serum cytokine levels were stable during the observation period (Figure 3). Interleukin-6 (Figure 3(e)) was the only cytokine that changed significantly (*p* values < 0.05) during the course of anti-TNF treatment. The baseline level was 20 pg/mL and it fell to 4.5 pg/mL at 3 months after treatment initiation and remained at this level for the rest of the study period.

#### 3.3.2. Matrix Metalloprotease (MMP)

**Figure 4:**
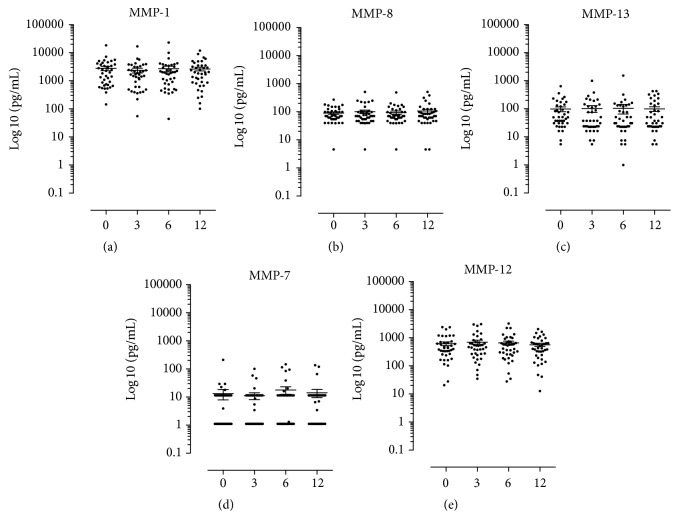
Matrix metalloprotease levels in serum from the RA patient cohort. (a) to (e) are scatter dot plots, where each dot represents one individual patient. The *x*-axes illustrate the four assessment months after infliximab treatment is initiated. The *y*-axes represent the concentrations in serum of their respective MMP in picograms per millilitre (pg/mL) on a log⁡10 scale. The horizontal lines in the dot plot illustrate the mean, and the vertical error lines represent ± standard error mean (SEM) in a paired *t*-test.

The serum levels of 5 MMPs were monitored, and none of the analysed MMPs changed significantly during the one-year study period (Figure 4). The average concentrations at baseline (with range) concentrations of the different MMPs were as follows: MMP-1 was 2781 pg/mL (145–18356 pg/mL), MMP-8 was 90.5 pg/mL (4.6–273.2 pg/mL), MMP-13 was 98.9 pg/mL (5.5–643.9 pg/mL), MMP-7 was 13.15 pg/mL (1.1–209.1 pg/mL), and MMP-12 was 620.7 pg/mL (20.6–2402 pg/mL).

#### 3.3.3. Vascular Endothelial Growth Factor (VEGF)

**Figure 5:**
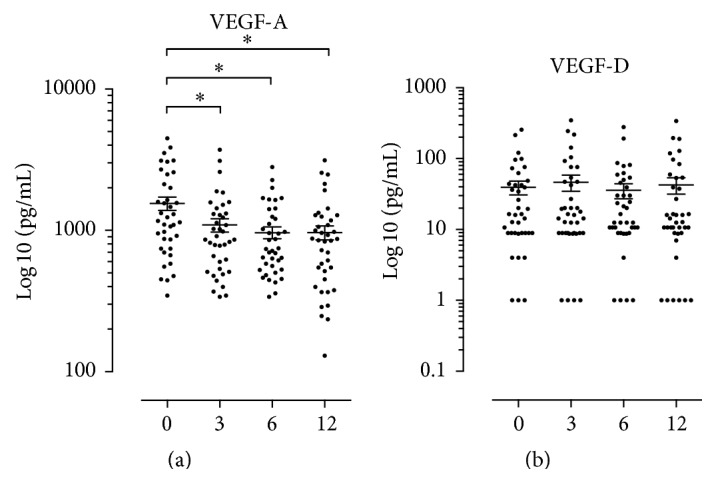
The VEGF-A and VEGF-D serum concentrations. The *x*-axes illustrate the four assessment months after infliximab treatment is initiated. The *y*-axes represent the concentrations in serum of their respective VEGF in picograms per millilitre (pg/mL) on a log⁡10 scale. The horizontal lines in the dot plot illustrate the mean, and the vertical error lines represent ± standard error mean (SEM) in a paired *t*-test.

The mean concentration of VEGF-A at baseline was 1553 pg/mL with a range of 345.9 to 4476 pg/mL, and after 12-month treatment the mean concentration of VEGF-A was 965.0 pg/mL with a range of 129.7 to 3128 pg/mL. VEGF-A showed a significant decrease (Figure 5(a)) from baseline compared with months 3, 6, and 12 (*p* values < 0.05).

The mean concentration of VEGF-D at baseline was 39.4 pg/mL with a range of 1.0 to 255.5 pg/mL, and 12 months after treatment the mean concentration of VEGF-D was 42.64 pg/mL with a range of 1.0 to 339.5 pg/mL. There was no significant change in VEGF-D when comparing baseline with 3, 6, or 12 months after treatment (Figure 5(b)). Additional results are presented in the supplementary material (in Supplementary Material available online at http://dx.doi.org/10.1155/2015/276815): distribution of ESR and CRP by gender and age (Supplementary Table 1) and frequencies of anti-CCP and rheumatoid factor (Supplementary Table 2); the clinical assessment of the RA patients (SWJ28, TEN28, ESR, CRP, MDGA-VAS, and PGA-VAS) during the study (Supplementary Figure 1); and the RA patients grouped by disease activity (Supplementary Figure 2) and the serum IgG-RF and anti-CCP titers during the study (Supplementary Figure 3).

## 4. Discussion

In this study, a cohort of 39 patients were selected from a prospective clinical open phase IV study which had a 3-year follow-up, where we focused on the first year of treatment. The aim was to examine the effect of infliximab treatment on disease status and progression in addition to several serological factors, which may be relevant serum markers for RA.

### 4.1. Decrease in Disease Activity

Already in 1999 Maini et al. showed clinical efficacy of infliximab treatment over 30 weeks in a phase III trial, where patients were treated with infliximab every 4 weeks concomitantly with methotrexate [12]. Our patients were in general in an advanced stage of disease with severe joint affection and tissue destruction when considering the clinical data (Figure 1). Collectively, the patient cohort disease activity and inflammation status decreased significantly, indicating that our patients benefited from the infliximab treatment. Several studies have been performed in an effort to ascertain anti-TNF-*α* treatment efficacy compared with DMARDs alone, and many report a positive effect [13–15]. The Sharp/van Der Heijde scores from the high-resolution X-ray imaging support that there was no significant change in joint damage collectively in the cohort (Figure 2), comparing baseline with 12 months after initiation of treatment. Only one patient showed extensive progression in joint damage, but this patient had severe joint damage already before entering the study.

### 4.2. Serum Markers

#### 4.2.1. Cytokines

Cytokines are important signaling molecules facilitating communication between cells of the immune system and useful as markers for ongoing immune reactions. We tested the serum level of a range of cytokines (IL-1*β*, IL-2, IL-4, IL-5, IL-6, IL-10, IL-12p70, IL-17A, IL-18, IFN-*γ*, TNF-*α*, and TNF-*β*); these cytokines represent a wide range of functional immune messengers.

Only IL-6 changed during the course of infliximab treatment, and it decreased significantly (Figure 3(e)). It has been suggested that IL-1*β* modifies IL-6 induced acute phase proteins in the liver by upregulation of CRP production [16]. This relationship between IL-6 and CRP might be one explanation for the apparent correlation between CRP and IL-6 reduction. Additionally, it has recently been proposed that baseline serum levels of IL-6, but not TNF-*α*, are a possible serum marker for disease activity in early RA [17].

Serum levels of TNF-*α* in healthy control sera have been reported to be lower than 6 pg/mL in a case study of a patient with large granular lymphocyte syndrome (LGL) [18]. Also, in a study of healthy subjects the mean TNF-*α* serum levels were reported to be between 30 and 40 pg/mL depending on age [19]. Our patients had an average of 1.8 pg/mL TNF-*α* at the start of the study, and the serum levels rose slightly to 7.3 pg/mL after 12 months, but this increase was not statistically significant.

#### 4.2.2. Matrix Metalloproteases

MMPs belong to a group of zinc-dependant endopeptidases involved in catalytic breakdown of extracellular matrix (ECM) proteins. Additionally, these enzymes serve important functions in tissue formation and remodelling, growth, wound healing, and maintenance. MMPs may contribute to tissue damage and break down if not properly regulated, and this is partly controlled by MMP inhibitors called Tissue Inhibitors of Metalloproteases (TIMPs).

We have tested MMPs that target collagen (MMP-1, MMP-8, and MMP-13), gelatin, fibronectin, and proteoglycans (MMP-7) and elastin and basement membrane components (MMP-12) [20]. We did not observe any change in MMPs levels, and they remained low during the study. This finding supports that inflammatory responses or joint erosion is not significantly increased during the study period.

Serum levels of MMP-3 in particular have previously been found to correlate with disease activity in RA and MMP-9 in systemic lupus erythematosus (SLE), which is another rheumatic disease [21, 22]. MMP-3 and MMP-9 have also been found to contribute to inflammatory conditions in the skin [20]. It has also been reported that infliximab reduces MMP-3 and MMP-9 levels in peripheral blood monocytes in patients with active RA [23]. Since we did not observe any significant disease progression in this study, these MMPs may also be stable in our cohort.

#### 4.2.3. Vascular Endothelial Growth Factors

We observed a weak decrease in serum concentration of VEGF-A throughout the study period (Figure 5(a)). Increased VEGF levels have been shown to induce TNF and IL-6 production [24, 25], which may promote inflammatory reactions. There may be a mutual and perhaps synergistic relationship between VEGFs and TNF/IL-6, as we observe a decrease in both IL-6 and VEGF-A in patients undergoing anti-TNF treatment, and with concurrent stable levels of joint destruction.

Vascular endothelial growth factors (VEGFs) consist of a family of cytokines and growth factors and are generally produced by cells in tissue that is oxygen depleted [26]. They mainly target endothelial cells via receptors (VEGF-R) and induce angiogenesis, that is, formation of new blood vessels. Many types of cells are capable of VEGF production and secretion, and they may also stimulate different cells, not only endothelial cells.

By inducing angiogenesis, VEGF is an important factor in facilitating cell adhesion and inflammation by promoting cell adhesion molecules and, in turn, in the recruitment of mononuclear cells like macrophages to target tissues [27]. VEGFs have been associated with development of joint destruction in RA, and it has been postulated that the destruction is mediated through the expansion of synovial vasculature [28–31].

Additionally, fibroblasts are reportedly stimulated by cytokines such as IL-1 and TNF-*α* to produce VEGF [25], which in turn can lead to increased proliferation of endothelial cells and vascularisation. VEGF has previously been associated with disease activity in RA and increased vascularisation may lead to increased leukocyte migration and mediate inflammation via this mechanism. VEGF has also been reported to correlate with erythrocyte sedimentation rate (ESR) and to contribute to the production of TNF and IL-6.

In conclusion, based on the various assessments and laboratory measurements performed in the clinic and the laboratory, we have shed some light on underlying features of the serology in rheumatoid arthritis. The study population showed a significant decrease in disease activity, indicating that anti-TNF treatment is efficacious despite long duration of RA. We observed no change in serum concentrations of many proinflammatory cytokines, TNF-*α*, and matrix metalloproteases during treatment. Nevertheless, we found statistically significant changes in serum levels of IL-6 and VEGF-A varying with disease activity. The clinical significance of this finding has to be further elucidated and confirmed in larger clinical trials before being utilized in a clinical setting.

## Supplementary Material

Additional results are presented in the supplementary material. Distribution of ESR and CRP by gender and age (Supplementary Table 1) and frequencies of anti-CCP and rheumatoid factor (Supplementary Table 2). The clinical assessment of the RA patients (SWJ28, TEN28, ESR, CRP, MDGA-VAS and PGA-VAS) during the study (Supplementary figure 1). The RA patients grouped by disease activity (Supplementary figure 2) and the serum IgG-RF and anti-CCP titers during the study (Supplementary figure 3).

## Figures and Tables

**Table 1:** Demographic data for the rheumatoid arthritis patient cohort.

Patient number^1^	Age of inclusion	RA duration^2^	Gender (M, F)^†^	Seropositive^3^		MTX dose^4^	PRD dose^5^	IFX dose^6^
	(years)	(years)		Waaler	Anti-CCP		(mg)	
101	60	5	F	**+**	**+**	10	7,5	300
102	45	14	F	**+**	**+**	5	2,5	300
106	50	22	M	**+**	**+**	7,5	0	300
107	49	4	F	**+**	**+**	7,5	5	300
108	60	10	F	**+**	**+**	10	2,5	200
110	59	6	F	**+**	**+**	7,5	0	200
111	57	6	F	**+**	**+**	15	2,5	200
112	57	15	F	**−**	**−**	5	7,5	400
113	64	5	F	**+**	**+**	10	0	200
116	66	1	F	**−**	**−**	10	7,5	300
121	54	11	F	**−**	**−**	2,5	7,5	200
125	48	6	M	**+**	**+**	2,5	0	300
126	35	8	M	**+**	**−**	15	10	300
127	57	3	M	**+**	**+**	20	7,5	300
130	68	11	M	**+**	**+**	15	0	300
131	71	21	M	**+**	**+**	7,5	10	300
132	56	3	F	**+**	**+**	15	7,5	200
133	30	11	F	**+**	**+**	10	5	300
134	54	18	M	**+**	**+**	10	5	300
135	56	13	M	**−**	**−**	15	5	400
136	52	7	F	**+**	**+**	7,5	5	200
137	21	3	M	**−**	**−**	15	5	300
138	56	24	F	**+**	**+**	20	5	300
139	59	13	M	**+**	**+**	15	10	200
201	58	7	M	**+**	**+**	15	5	300
205	61	2	F	**−**	**+**	15	0	200
207	62	29	F	**+**	**+**	12,5	2,5	200
215	34	14	F	**+**	**+**	15	10	200
218	74	6	F	**+**	**+**	20	5	200
223	44	15	F	**+**	**+**	15	0	200
233	59	2	F	**+**	**+**	7,5	0	200
302	64	5	F	**−**	**−**	7,5	10	200
303	74	21	F	**−**	**−**	7,5	5	300
304	57	21	F	**−**	**+**	7,5	0	300
305	57	26	M	**−**	**+**	15	0	300
306	36	13	M	**+**	**+**	7,5	0	300
307	56	18	M	**+**	**+**	20	0	300
308	35	13	F	**−**	**+**	10	0	300
309	43	21	M	**+**	**+**	12,5	5	200

^1^Ref. no.: internal patient number, 1xx: Haugesund, 2xx: Bergen, and 3xx: Førde. ^2^Estimated duration of rheumatoid arthritis disease prior to infliximab treatment. ^3^Clinical data before treatment (baseline), +: seropositive, −: seronegative, ^4^MTX: methotrexate, ^5^PRD: prednisolone, ^6^IFX: infliximab, ^†^F: female, and M: male.

**Table 2:** Summary of the RA patient cohort data. Age and gender distribution and mean pharmacological doses.

Variable	
Number of patients	39
Mean age ± SD (range)	53 ± 12 (21–74)
Gender ratio (F : M^1^)	24 : 15
RA duration ± SD (range)	11 ± 8 (1–29)
RF-positive (*n*/%)	28/72
Anti-CCP positive (*n*/%)	31/80
Mean methotrexate dose ± SD (range)	11 ± 5 (2.5–20)
Mean infliximab dose ± SD (range)	264 ± 58 (200–400)
Mean prednisolone dose ± SD (range)	4 ± 4 (0–10)

^1^Female to male ratio.
