# Supplementary material for: Serum Markers in Rheumatoid Arthritis: A Longitudinal Study of Patients Undergoing Infliximab Treatment
Source: Int J Rheumatol. 2015 Dec 16;2015:276815. doi: 10.1155/2015/276815 (PMC4695666; doi:10.1155/2015/276815)
Supplement: Supplementary file 1 — Additional results are presented in the supplementary material. Distribution of ESR and CRP by gender and age (Supplementary Table 1) and frequencies of anti-CCP and rheumatoid factor (Supplementary Table 2). The clinical assessment of the RA patients (SWJ28, TEN28, ESR, CRP, MDGA-VAS and PGA-VAS) during the study (Supplementary figure 1). The RA patients grouped by disease activity (Supplementary figure 2) and the serum IgG-RF and anti-CCP titers during the study (Supplementary figure 3). [file 276815.f1.pdf]

Supplementary Tables 1-2

Supplementary Table 1. Distribution of ESR (A and B) and CRP (C and D) by gender and age.

A) Distribution of ESR in mm per hour in the male population of the cohort.

**Men**	Assessment time points			
n = 15	**0**	**3**	**6**	**12**
Mean	29.0	15.7	26.0	29.1
SD†	21.2	14.2	15.9	17.1
Min-max	2-81	2-60	8-59	10-59
Positive < 50 years	2	0	4	4
Positive > 50 years	8	4	6	7

B) Distribution of ESR in mm per hour in the female population of the cohort.

**Women**	Assessment time points			
n = 24	**0**	**3**	**6**	**12**
Mean (mg/L)	40.1	21.2	22.5	29.8
SD	24.0	18.4	17.4	25.7
Min-max	8-105	5-80	5-86	7-90
Positive < 50 years	4	2	0	1
Positive > 50 years	12	3	4	6

C) Distribution of CRP in mg per litre in the male population of the cohort.

**Men**	**Assessment time points**			
n = 15	**0**	**3**	**6**	**12**
Mean	25.1	7.8	12.3	15.8
SD	13.9	14.0	10.4	14.8
Min-max	0-51	0-42	0-28	0-46
Positive > 5 mg/L	14	5	11	10

† SD = Standard deviation

D) Distribution of CRP in mg per litre in the female population of the cohort.

**Women**	**Assessment time points**			
n = 24	**0**	**3**	**6**	**12**
Mean	41.0	10.3	7.9	18.4
SD	30.2	15.8	17.2	36.2
Min-max	7-135	0-60	0-80	0-164
Positive > 5 mg/L	24	10	9	13

Supplementary Table 2. Rheumatoid factor and anti-CCP positive RA and SS patients.

A) Sero-positive RA and SS patients at baseline, 3, 6 and 12 months after initiation of treatment.

	RA patients (n=39)				SS patients (n=134)
**Assessment time points**	**0**	**3**	**6**	**12**	**N/A‡**
RF Positive (**n**/%)	**6**/15	**7**/18	**7**/18	**7**/18	**52**/39
Anti-CCP2 Positive (**n**/%)	**31**/80	**30**/77	**30**/77	**28**/72	N/A
Double positive (**n**/%)	**4**/10	**4**/10	**4**/10	**5**/13	N/A

B) Number of sero-positive patients compared with initial analysis in the clinic at baseline.

	**Thesis** (n=39)	**Clinic** (n=39)
RF-positive (**n**/%)	**6**/15	**28**/72
Anti-CCP2 positive (**n**/%)	**31**/80	**31**/80
Double positive (**n**/%)	**4**/10	**27**/69

Supplementary Figures 1 - 5

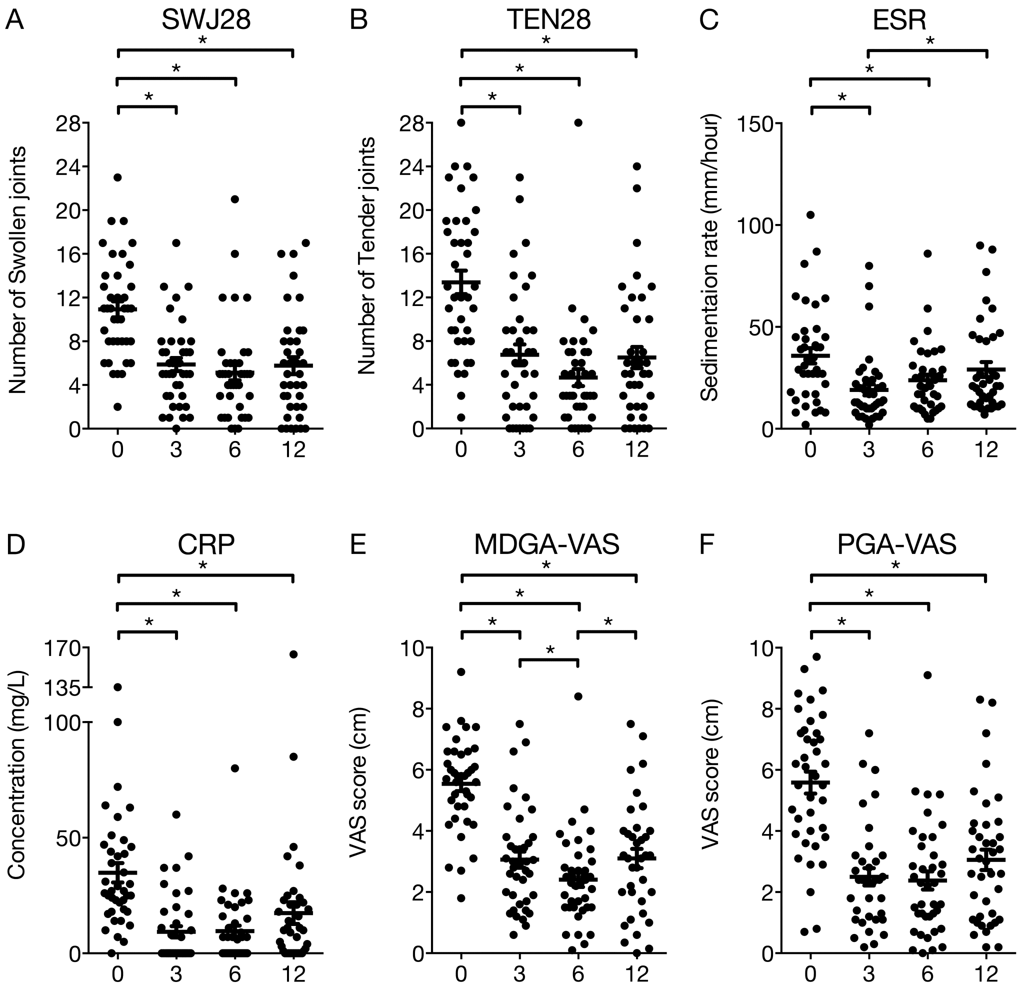

Supplementary Figure 1. Various clinical assessments show a significant decrease in disease activity Scatter dot plots where each dot represents one individual patient. The x-axes illustrate the four assessment time points indicated as 0, 3, 6 and 12 months after treatment is initiated. The horizontal lines in the dot plot illustrate the mean, and the vertical error lines are ± standard error mean (SEM) in a paired t-test. **A)** Number of swollen joints. **B)** Number of tender joints. **C)** Erythrocyte sedimentation rate, horizontal dotted line illustrates the highest reference value. **D)** C-reactive protein concentration., horizontal dotted line illustrates the reference concentration. **E)** Visual Analogue Scale scores for individual patients, assessed by rheumatologists. **F)** Visual Analogue Scale scores, subjectively assessed by the individual patient.

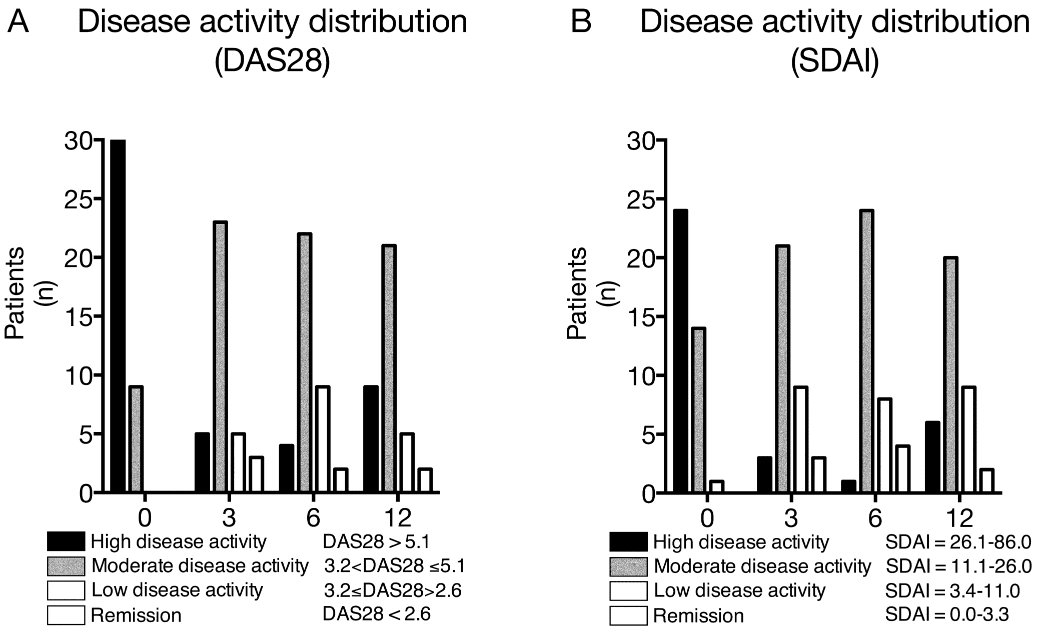

Supplementary Figure 2. Distribution of in RA patients into grouped disease activity using the DAS28 and SDAI scores. A) Disease activity distribution for SDAI during the study period; B) Disease activity distribution for DAS28 during the study period. The y-axes represent the number of patients of either disease activity; i) high; ii) moderate; iii) low; and iv) patients who are considered in remission. The x-axes illustrate the four assessment time points indicated as 0, 3, 6 and 12 months after treatment is initiated.

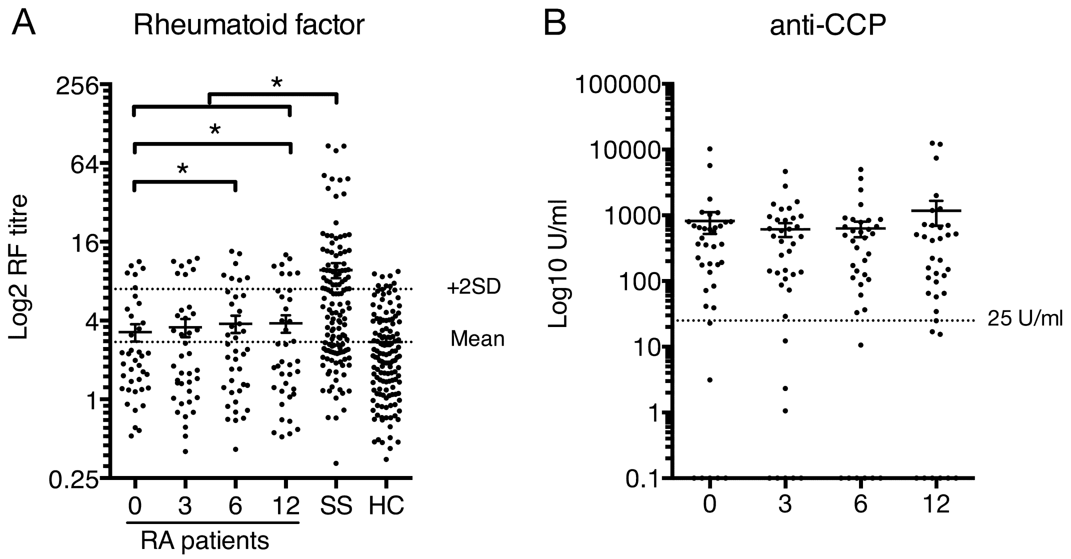

Supplementary Figure 3. IgG RF and anti-CCP in rheumatoid arthritis patients. Figures A and B are scatter dot plots, where each dot represents one individual patient. The x-axes illustrate the four assessment months after infliximab treatment is initiated. A) Showing the IgG-RF titers in RA patients, Sjögrens' Syndrome patients (SS) and blood donors/healthy controls (HC). The mean and the mean+2SD values of the healthy controls are indicated by a dotted lines, indicating sero-positive (above) and negative (below) titers. B) The levels of anti-CCP in the RA patients over the study period given as units per milliliter (U/ml) on a log10 scale. The cut-off value of 25U/ml is indicated by a dotted line.
